# Effects of unilateral and bilateral contrast training on the lower limb sports ability of college basketball players

**DOI:** 10.3389/fphys.2024.1452751

**Published:** 2024-11-22

**Authors:** Tianyu Duan, Zongwei He, Jing Dai, Lin Xie, Yuer Shi, Lunxin Chen, Junyi Song, Guoxing Li, Wenfeng Zhang

**Affiliations:** ^1^ Digitalized Performance Training Laboratory, Guangzhou Sport University, Guangzhou, Guangdong, China; ^2^ Graduate School, Guangzhou Sport University, Guangzhou, Guangdong, China; ^3^ Sports Training Institute, Guangzhou Sport University, Guangzhou, Guangdong, China; ^4^ Guangdong Provincial Key Laboratory of Human Sports Performance Science, Guangzhou, Guangdong, China

**Keywords:** unilateral training, bilateral training, contrast training, basketball, sports ability

## Abstract

**Objective:** The purpose of this study was to compare the impact of unilateral (U) and bilateral (B) contrast training on lower limb explosiveness, agility, and balance in college basketball athletes.

**Methods:** Twenty male college basketball players were randomly assigned to either a unilateral group (U, n = 10) or a bilateral group (B, n = 10). Both groups underwent an 8week strength training program, with sessions held twice a week. The unilateral group performed six Bulgarian split squats and ten reverse lunge jump squats, while the bilateral group performed six barbell rear squats and ten double-leg vertical jumps. To comprehensively assess the training effects, the study utilized one-repetition maximum (1RM), countermovement jump (CMJ), 20m sprint, and single-leg hop tests to evaluate explosive power; the 505 and *t*-test to assess change-of-direction ability; and the Y-balance test (YBT) to evaluate dynamic balance. Paired sample t-tests were used to evaluate within-group changes, and a 2 (pre- and post-) × 2 (experimental and control groups) repeated measures analysis of variance (ANOVA) was used to assess between-group differences.

**Results:** Within-group comparisons indicated that both unilateral and bilateral contrast training significantly improved all performance metrics. Between-group comparisons revealed that bilateral training was superior to unilateral training in improvements in 1RM and CMJ (*p* > 0.05) (growth rate of 1RM: B: 8.4%, U: 5.15%; growth rate of CMJ: B: 15.63%, U: 6.74%). Unilateral training showed greater improvements in the 20m sprint, dominant leg single-leg hop, YBT left, and YBT right (*p* > 0.05) (growth rate of 20m sprint: B: 5.43%, U: 10.41%; growth rate of advantage foot touch high: B: 4.56%, U: 9.35%; growth rate of YBT left: B: 3.77%, U: 8.53%; growth rate of YBT right: B: 4.72%, U: 13.8%). Unilateral training also significantly outperformed bilateral training in non-dominant leg single-leg hop, *t*-test, 505 left, and 505 right improvements (*p* < 0.05).

**Conclusion:** Unilateral contrast training may offer advantages for enhancing change-of-direction ability and explosive power in the non-dominant leg, and it may also provide benefits for improving short-distance sprinting ability, explosive power in the dominant leg, and dynamic balance. In contrast, bilateral contrast training appears to be more effective for enhancing bilateral explosive power and may be more advantageous for increasing maximal strength.

## 1 Introduction

The sport of basketball imposes significant demands on athletes' physical capabilities, particularly in terms of lower limb explosiveness, confrontational strength, and body control. The execution of basketball maneuvers is predominantly facilitated by the lower limbs, thereby necessitating athletes to have outstanding lower limb athletic abilities (Stojanović et al., 2018). During a high-level basketball match, athletes are observed to perform an average of 37–51 rebounding actions (Ben Abdelkrim, El Fazaa, and El Ati, 2007). They change direction every 1–3 s (Scanlan et al., 2015) and engage in 55–105 sprints within a 21–39s timeframe (Conte et al., 2015). The proficient execution of these actions requires a high level of lower limb explosive power and agility (Hammami et al., 2018). Moreover, the stable landing following technical moves and competitive engagements highlights the critical role of dynamic balance (Montgomery, Pyne, and Minahan, 2010).

Contrast training, an amalgamation of resistance and plyometric exercises, serves as an effective strategy to elevate athletic prowess, particularly in sports that demand acute lower limb capabilities (Carter and Greenwood, 2014). Studies have established the significant role of contrast training in augmenting lower limb explosiveness and agility in team sports such as basketball, soccer, and rugby (Hou and Li, 2022; Thapa et al., 2023; Zhou and Zhang, 2017). However, it is noted that the impact of various modalities of contrast training on athletic enhancement varies, suggesting a nuanced approach to training regimens is essential.

Combining unilateral and bilateral training with contrast exercises represents two distinct modalities within contrast training, each yielding unique outcomes. Unilateral training refers to the practice conducted using a single limb movement pattern (Lin and Ye, 2022). The cross-transfer theory posits that after resistance training of one limb, there may be an improvement in strength and/or skill in the contralateral, untrained limb (Hendy, Spittle, and Kidgell, 2012). The primary significance of unilateral training lies in its ability to independently stimulate the target muscles, facilitating more precise muscle conditioning in specific areas (Zhang et al., 2023). Some studies have reported that unilateral training can more effectively enhance strength, jumping ability, sprinting performance, and balance (Ramírez-Campillo et al., 2015; Bogdanis et al., 2019; Gonzalo-Skok et al., 2017). A research on the effects of unilateral and bilateral training on muscle hypertrophy and athletic performance found that unilateral training significantly improves lower limb muscle quality and functional performance (Núñez et al., 2018). A study noted that unilateral training is more effective than bilateral training in enhancing unilateral explosive power (Lin and Ye, 2022). However, other research suggests that bilateral training may offer advantages over unilateral training in terms of improving bilateral jumping ability and maximal strength (Makaruk et al., 2011; Mudlo, 2014; Bogdanis et al., 2019). Given the ongoing debate regarding the differential effects of unilateral and bilateral training, and the scarcity of studies combining unilateral and bilateral training with contrast exercises, this study aims to explore an optimal and efficient training method to enhance lower limb athletic capabilities in basketball players. By comparing the impact of unilateral and bilateral contrast training on lower limb explosiveness, agility, and balance in college basketball athletes, the study seeks to add new knowledge on this topic regarding which training can be most effective for improving lower limb athletic performance. The hypothesis of this study is that unilateral contrast training may yield better results in enhancing lower limb explosive power, agility, and dynamic balance compared to bilateral contrast training.

## 2 Materials and methods

### 2.1 Subject

The sample size was calculated using G-power, with ES = 0.5, α err prob = 0.05, and power (1- β err prob) = 0.8. The results showed that a minimum of 18 samples were required. To prevent sample size loss, an increase of 10% was made on this basis, and the final recruitment number was determined to be 20 people. Participants were selected from the university basketball team. Inclusion criteria for the experimental participants were as follows: 1) athletes with a sports classification of at least second level or with training experience of at least 3 years; 2) they could perform a maximum squat lift equivalent to 1.5 times their body weight; 3) they had no history of injury within the past 6 months; 4) they had experience with resistance training; 5) the athletes were in good health. The basic information of the 20 participants, including height, weight, age, and years of athletic activity, was statistically organized using Microsoft Excel. Participants were then randomly assigned to either a unilateral group (n = 10) or a bilateral group (n = 10) using a stratified randomization method based on their positions in basketball matches. Table 1 presents the basic characteristics of the participants, with no significant baseline differences observed between the two groups. This study adheres to the Declaration of Helsinki by the World Medical Association. All participants voluntarily took part in the experiment and provided written informed consent form. This study has been approved by the Ethics Committee (ID Number:2023LCLL-79) and also registered with the Chinese Clinical Trial Registry (Registration number: ChiCTR2400083068).

**TABLE 1:** Sample characteristics.

	U (n = 10)	B (n = 10)	T	*p*
Height (cm)	183.20 ± 6.82	183.00 ± 5.86	−0.07	0.945
Body Mass (kg)	77.90 ± 5.90	76.20 ± 8.70	−0.511	0.616
Standing touch height (cm)	236.40 ± 10.74	235.10 ± 11.14	−0.266	0.794
Age (year)	19.90 ± 1.45	20.80 ± 1.14	1.546	0.140
Sport experience (years)	4.80 ± 1.14	5.00 ± 1.05	0.408	0.688

U: unilateral group; B: bilateral group.

### 2.2 Procedure and evaluation

**FIGURE 1:**
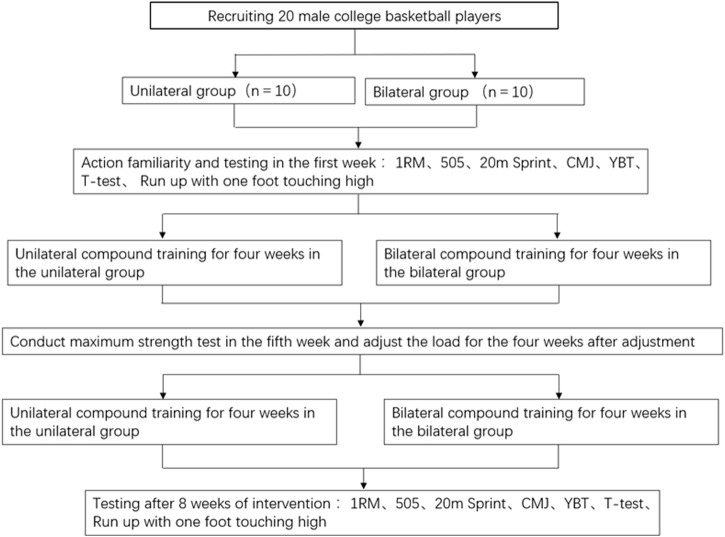
Experimental flowchart.

The study lasted for 10 weeks, including a 2-week period for initial and final assessments (pre- and post-tests) and an 8week experimental intervention phase, with training sessions conducted twice weekly on Tuesdays and Thursdays. During the first week, participants were introduced to the exercises and underwent a thorough series of performance tests. The first testing session included assessments of the 505 test, the one-repetition maximum (1RM) squat, and the 20m sprint. The second session involved tests of the countermovement jump (CMJ), the sprinted single-leg reach, the *t*-test, and the Y Balance Test (YBT). Before administering the tests, each participant performed the same standardized warm-up consisting of running exercises; strength, plyometrics, and balance exercises; and running exercises since it is known that warm-up can affect performance (Patti et al., 2022). At the onset of each testing session, participants were thoroughly briefed on the procedures to ensure they were well-acquainted with the testing protocols. At the midpoint of the experimental period, specifically during the fifth week, a maximal strength test was conducted, and the training loads were adjusted accordingly. One week after the intervention ended, the final assessments were conducted, and data collection was finished. Participants were instructed to wear the same footwear and attire during both pre- and post-intervention testing periods to control for variables that could affect the results (Figure 1 Experimental flowchart).

### 2.3 Training program

The intervention took place at the school’s fitness center. To minimize the influence of confounding factors, no lower limb strength training was scheduled during the formal experimental period. After the warm-up, the unilateral and bilateral groups underwent the experimental intervention simultaneously. Throughout the entire experiment, both groups of subjects were kept consistent in terms of training frequency, number of sets, duration, venue arrangements, training content, and load, with differences only in the training methods employed. During the intervention, participants were not permitted to engage in any other form of physical training. The intervention was supervised and implemented by two coaches to ensure rigor and accuracy.

The unilateral contrast training group engaged in five sets per training session, with each exercise being performed unilaterally. A set was completed when both legs had finished the prescribed repetitions. Each set consisted of six Bulgarian split squats per side, followed by ten reverse lunge plyometric jumps. The bilateral contrast training group also completed five sets per session, with all exercises performed bilaterally. Each set included six barbell squats behind the neck, succeeded by ten bilateral vertical jumps.

To ensure equivalent training loads for each leg between the two groups, the pressure exerted by the support leg during the Bulgarian split squat was set to 85% of the total load (the sum of body weight and additional resistance). Consequently, the training load for the unilateral leg exercises was calculated as follows: for the barbell squat, each leg’s load (F1) was determined by the equation F1 = (N% of 1RM squat weight + body weight)/2, where N% represents the percentage of the one-repetition maximum (1RM) used in the exercise. The load for the front leg of the Bulgarian split squat (F2) was calculated using the equation F2 = (Fx + body weight) * 85%, where Fx is the load on the front leg during the Bulgarian split squat. Given that the loads F1 and F2 were made equivalent (F1 = F2), the weight for the Bulgarian split squat (Fx) was derived from the equation Fx = (N% of 1RM + body weight)/(2 * 85%) (Yang, 2016) (Table 2).

**TABLE 2:** Training intervention.

	Practice content	Load intensity	Number of sets	Training volume	Rest period
U	Bulgarian Squat + Rear Leg Elevated Split Squat Jump	[(85%1RM squat + body weight) ÷ (2% × 85%) - body weight]+ self-weight hind leg raise split leg squat jump	5	6 repetitions +10 repetitions/side	each movement has a 15-s rest, with a 3min rest between sets
B	Squat + Double Jump	85%1RM + self-weight double leg longitudinal jump	5	6 repetitions +10 repetitions	each movement has a 15s rest, with a 3min rest between sets

U: unilateral group; B: bilateral group.

### 2.4 Test index

#### 2.4.1 Back squat 1RM test

The 1RM back squat test was performed according to the guidelines specified in the “National Strength and Conditioning Association (NSCA) - Certified Strength and Conditioning Specialist (CSCS)” manual (Haff and Triplett, 2021). Before the squat, participants performed standardized warm-up exercises. They then completed five repetitions at an estimated 50% of their one-repetition maximum (1RM), three repetitions at approximately 70% of their 1RM, and two repetitions at about 80% of their 1RM. Following this, subjects attempted to determine their 1RM with progressively increasing loads. Participants were required to squat until their thighs were parallel to the ground, with a maximum of five attempts allowed. The weight successfully lifted on the last attempt was recorded as the 1RM. A 2min rest period was provided between each attempt. For safety, at least two personnel were present to assist (Earle, 2012).

#### 2.4.2 20m sprint test

The 20m sprint test was conducted using a wireless photoelectric timing system (Brower Timing Systems, Salt Lake City, United States). Using Brower Timing Systems to test short-distance sprints has high reliability (Bond, Willaert, and Noonan, 2017). The photoelectric sensors were positioned at the starting and finishing lines, 20m apart, and adjusted to a height of 0.8 m above the ground, level with the participants' hips. Upon hearing the command “start,” participants sprinted with maximal effort and were instructed to decelerate only after crossing the photoelectric beam at the finish line, completing the prescribed sprint distance. Each participant completed two trials, with a 2min rest period between trials. The best performance from the two attempts was recorded and used for statistical analysis (Hammami et al., 2018).

#### 2.4.3 CMJ test

The CMJ test was performed using a jump mat (Fusionsport, Perth, Australia). Research indicates that using Fusionsport to test the height of countermovement jump has high reliability (Kobal et al., 2017). During the test, participants stood with their hands by their sides on an electronic jump mat and, after a rapid squat, they performed a maximal effort vertical jump with arm swing, landing back within the confines of the mat. The jump height was the measured variable. Each participant completed three trials, and the highest jump was selected for statistical analysis. There was approximately 30 s of rest time between each trail (Chang et al., 2022).

#### 2.4.4 One-footed running high jump

The sprinted single-leg reach test was performed using a reach tester (AIAY, Dongguan, China). Participants executed the test by running up to the reach tester from a distance of 3–5 m and performing a jump-off using either their dominant or non-dominant leg. Upon jumping, participants were instructed to reach up with their dominant arm to touch a movable slider as high as possible at the apex of their jump. The final score recorded was the vertical distance between the ground and the highest point reached by the slider touched by the participant’s dominant hand. After completing two trials with the dominant leg, participants performed two trials with the non-dominant leg. The best result from these four attempts was selected for analysis. There was 1 min interval between each test (Wang, 2022).

#### 2.4.5 505 change-of-Direction Speed Test

The 505 Change-of-Direction Speed Test was conducted using a wireless photoelectric timing system (Brower Timing Systems, Salt Lake City, United States). The device shows high reliability in testing agility (Brown, 2012). A distance of 15 m was measured, with the wireless photoelectric sensor positioned at the 10m mark and a clear marker set at the 15m point to provide a distinct target for athletes as they approached the turn. Participants sprinted 15 m on a rubberized track and, upon reaching the 15m mark, executed a 180° turn to sprint an additional 5 m to the finish line photoelectric beam. Any trial in which the participant changed direction before reaching the turn line was disregarded. The test was completed twice for both the left and right legs (i.e., executing the 15 m turn using either the left or right foot to touch the turn line), with a 2min rest period between each trial. The fastest time from the four attempts was used for data analysis (Lockie, Dawes, and Jones, 2018).

#### 2.4.6 *t*-test

**FIGURE 2:**
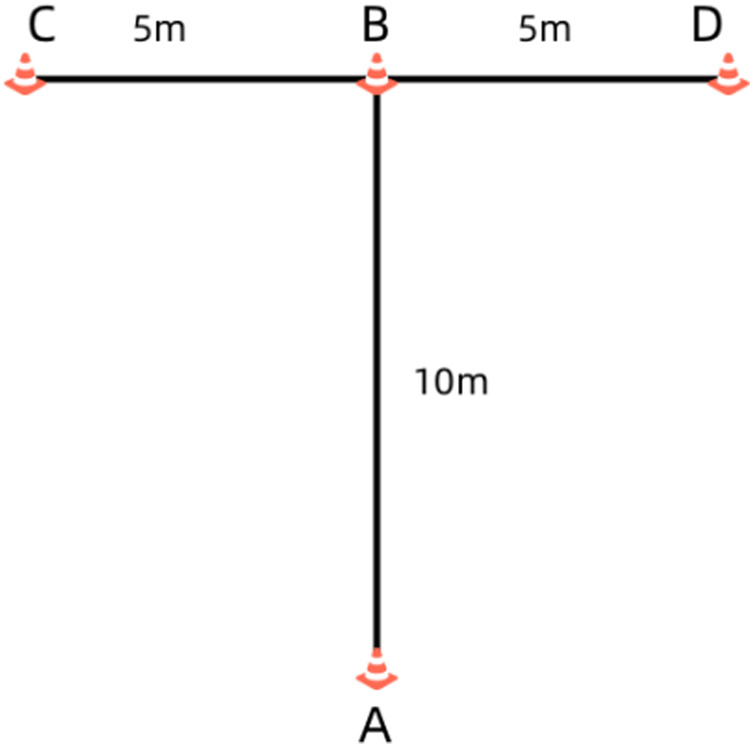
t-test.

The *t*-test was conducted using a wireless photoelectric timing device (Brower Timing Systems, Salt Lake City, United States) and four marker cones. The cones were positioned at points A, B, C, and D, arranged in a “T”shape, with the wireless photoelectric sensor located at point A. Upon hearing the signal, participants initiated the test from point A, sprinting forward 10 m to point B and touching the marker with their right hand. They then faced forward and, without crossing their steps, side-shuffled laterally 5 m to the left, touching the marker at point C with their left hand. Subsequently, they quickly side-shuffled to point D, touching the marker with their right hand, followed by a lateral shuffle back to point B. Finally, participants retreated by running backward to the starting cone at point A, completing the test. Each participant performed the test twice, with the fastest trial used for analysis. The time interval between two tests is 5 min (Kadlubowski et al., 2019) (Figure 2 *t*-test).

#### 2.4.7 YBT

The YBT was performed using a Y Balance Tester. Research has indicated that the Y Balance Test exhibits excellent inter-rater reliability (Alshehre et al., 2021). Prior to the commencement of the test, the lower limb length of each participant was measured, which is defined as the distance from the anterior superior iliac spine to the center of the medial malleolus of the ankle. Participants stood on one foot, barefoot, with their toes positioned behind the red line on the Y Balance Tester. They placed their hands on their waist and maintained their balance while extending the opposite leg to push the testing plate slowly and continuously in three different directions: anterior, posterolateral, and posteromedial. After three practice trials to familiarize themselves with the procedure, participants then undertook the formal testing. Each lower limb was tested three times in each of the three directions, with a 15s interval between each test. The best performance for each direction was recorded, with data accuracy to the nearest 0.1 cm (Plisky et al., 2009).

### 2.5 Statistical analyses

All variables were subjected to a normality test using the Kolmogorov-Smirnov statistic, and the homogeneity of variances was determined using Levene’s test. Data within the text are presented as mean and standard deviation (Mean ± SD). Analysis of the data was performed using JASP 0.18.1.0, employing a 2 (pre- and post-comparison) × 2 (experimental group and control group) repeated measures analysis of variance (ANOVA) to assess the differences in changes between groups. Inter-group differences were measured using the eta squared (η^2^) statistic to determine the effect size of the intervention, with the criteria for classification being small (0.01 ≤ η^2^ < 0.059), medium (0.059 ≤ η^2^ < 0.138), or large (η^2^ ≥ 0.138). Intra-group differences were assessed using paired-samples T-tests, with the effect size (ES) used to measure the magnitude of differences between pre- and post-tests within each group. The criteria for determining the effect size (ES) are as follows: small effect (0.2), medium effect (0.5), and large effect (0.8). A comparison of the subjects' baseline characteristics and the inter-group differences in Rating of Perceived Exertion (RPE) was analyzed using independent samples T-tests. *p* < 0.05 indicates a statistically significant difference.

## 3 Result

Intergroup comparisons revealed that unilateral contrast training significantly outperformed bilateral contrast training in the improvements of disadvantage foot touch high, *t*-test, 505 test left, and 505 test right (*p* < 0.05). The Cohen’s d value for the between-group comparisons indicate medium to large effects (disadvantage foot touch high: 0.799; *t*-test: 1.584; 505 test left: 1.881; 505 test right: 1.314). The magnitude of these Cohen’s d value further supports the significant differences observed between the groups. Additionally, unilateral contrast training demonstrated a more pronounced advantage in the enhancements of the 20m sprint, dominant leg sprinted reach, YBT left, and YBT right, with the following percentage improvements: 20m sprint (Bilateral: 5.43%, Unilateral: 10.41%), dominant leg sprinted reach (B: 4.56%, U: 9.35%), YBT left (B: 3.77%, U: 8.53%), and YBT right (B: 4.72%, U: 13.80%). Conversely, bilateral contrast training showed a greater advantage in the improvements of 1RM and CMJ, with the following percentage improvements: 1RM (B: 8.4%, U: 5.15%) and CMJ (B: 15.63%, U: 6.74%). (Table 3).

**TABLE 3:** Changes before and after the experiment.

Index	Control group(B)					Experimental group(U)			Inter-group comparison			
			*p*	Cohend’s d	Improvement in percentage		*p*	Cohend s d	Improvement in percentage	*p*	η^2^_p_	Cohen’s d
1RM	Pre	125 ± 8.50	<0.001	1.495	8.4%	126 ± 8.10	0.002	0.926	5.15%	0.738	0.006	0.142
	Post	135.5 ± 5.99				132.5 ± 4.86						
CMJ	Pre	55.34 ± 4.99	<0.001	1.580	15.63%	54.84 ± 7.39	0.027	0.675	6.74%	0.211	0.085	0.543
	Post	64.00 ± 3.17				58.54 ± 5.53						
20m sprint	Pre	3.31 ± 0.10	0.007	1.374	5.43%	3.36 ± 0.13	<0.001	2.565	10.41%	0.525	0.023	0.225
	Post	3.12 ± 3.01				3.01 ± 0.16						
Advantage foot touch high	Pre	76.70 ± 6.16	0.020	0.587	4.56%	75.90 ± 5.43	<0.001	1.191	9.35%	0.699	0.009	0.168
	Post	80.20 ± 5.85				83.00 ± 6.36						
Disadvantage Foot Touch High	Pre	65.30 ± 2.67	0.039	0.284	1.99%	64.00 ± 6.13	0.001	2.450	17.5%	0.037	0.219	0.799
	Post	66.60 ± 3.24				75.20 ± 5.33						
*t*-test	Pre	11.79 ± 0.55	0.004	0.544	2.12%	11.40 ± 0.43	<0.001	2.024	8.16%	0.002	0.427	1.584
	Post	11.54 ± 0.47				10.47 ± 0.37						
505 left	Pre	3.22 ± 0.09	0.032	0.919	2.79%	3.24 ± 0.07	<0.001	5.083	14.81%	<0.001	0.607	1.881
	Post	3.14 ± 0.11				2.76 ± 0.10						
505 right	Pre	3.17 ± 0.08	<0.001	1.566	4.43%	3.14 ± 0.12	<0.001	3.571	10.54%	0.005	0.369	1.314
	Post	3.02 ± 0.08				2.81 ± 0.08						
YBT left	Pre	91.09 ± 4.30	0.007	0.821	3.77%	92.46 ± 2.84	<0.001	1.880	8.53%	0.051	0.196	0.854
	Post	94.54 ± 5.32				100.35 ± 3.94						
YBT right	Pre	86.98 ± 4.21	<0.001	0.742	4.72%	85.48 ± 5.72	<0.001	2.131	13.8%	0.344	0.050	0.424
	Post	91.0 ± 4.30				97.29 ± 7.34						

B: bilateral group; U: unilateral group.

## 4 Discussion

The findings of this study indicate that both unilateral and bilateral contrast training can enhance the explosive power, change-of-direction ability, and dynamic balance capabilities of college basketball players. The unilateral training group demonstrated greater advantages in improving change-of-direction skills, unilateral jumping ability, short-distance sprinting ability, and dynamic balance. In contrast, the bilateral training group showed a more significant advantage in enhancing bilateral explosive power and maximal strength.

### 4.1 Explosive power

Explosive power is a manifestation of rapid strength, referring to the ability of muscles, in which tension has already begun to increase, to overcome resistance at the fastest possible speed (Tian, 2000). Maximum strength, jumping ability, and rapid sprinting are all indicators that reflect the lower limb explosive power of basketball players (Feng, Zhou, and Wang, 2021; Wang, 2020; Cabrejas et al., 2023; Weng et al., 2022; Notarnicola et al., 2018; Hammami, Negra, et al., 2018; Hou and Li, 2022).

Maximum strength is the foundation for the development of explosive power, and its enhancement depends on two aspects: an increase in muscle cross-sectional area and an improvement in motor unit recruitment capacity (Chen, 2004). In this study, both the unilateral and bilateral contrast training groups showed a significant increase in maximum strength compared to pre-experimental levels, indicating that different contrast training methods are effective in enhancing maximum strength. However, no significant differences were observed between the two groups. This may be due to the fact that strength training typically results in an increase in muscle cross-sectional area, known as muscle hypertrophy. Over time, both unilateral and bilateral training can promote muscle protein synthesis and the growth of muscle fibers, thereby increasing strength (Botton et al., 2016). Additionally, the consistent training load between unilateral and bilateral contrast training may also be a reason for the lack of significant differences in training effects. In summary, both unilateral and bilateral contrast training can effectively improve the lower limb maximum strength of male college basketball players, with the bilateral contrast training group showing a slight advantage over the unilateral contrast training group, but the difference is not significant. In basketball games, players often need to sprint short distances, and short-distance sprinting can reflect the ability to accelerate quickly during horizontal rapid displacement. A significant change was observed post-experiment compared to pre-experiment in both groups, indicating that both unilateral and bilateral contrast training can enhance athletes' sprinting ability, which is consistent with the improvement in lower body maximum strength. This finding also supports a meta-analysis study that showed improvements in strength have a positive impact on short-distance sprint performance (Seitz et al., 2014). Additionally, it has been shown that the post-activation potentiation effect significantly outperforms conventional training in enhancing sprint performance (Zhang et al., 2024). Although no significant differences were observed post-experiment between the two groups, the unilateral contrast training showed a relatively greater advantage in improving sprinting ability. This may be because the Bulgarian split squats performed in the unilateral group have a similar unilateral force generation movement pattern to short-distance sprinting, which can more directly simulate and strengthen the specific muscle activities and neuromuscular coordination patterns during sprinting. Therefore, unilateral contrast training may have a more significant advantage in improving short-distance sprinting ability (Turner and Jeffreys, 2010).

In a basketball game, players perform around 50 jumps, both single and double-footed (predominantly double-footed) (Xu, Cheng, and Wang, 2011). Jump height, as an indicator of explosive power in the vertical direction, can reflect the impact of unilateral and bilateral contrast training on explosive power. The results show that contrast training in both modalities can improve athletes' jumping ability. Bilateral contrast training slightly outperforms unilateral contrast training in enhancing bilateral jumping ability, likely because the bilateral contrast training group involves bilateral movement patterns, which are similar to the CMJ, which also involves a two-foot takeoff. Unilateral contrast training is more effective in improving unilateral jumping ability (non-dominant leg); however, there is little difference between the two training groups in improving unilateral jumping ability (dominant leg). From the perspective of cross-transfer, training of one limb can promote strength gains in the contralateral limb, and this transfer is usually from the dominant to the non-dominant side. When training the dominant side, the non-dominant side also improves, hence the improvement in the non-dominant leg sprinted reach is more pronounced (Sun, 2021).

### 4.2 Change-of-direction ability

Both unilateral and bilateral contrast training have improved the change-of-direction ability of basketball players to varying degrees, with unilateral contrast training showing a significantly greater improvement in change-of-direction ability compared to bilateral contrast training. This may be attributed to the fact that change-of-direction movements often involve unilateral leg force generation and support. Unilateral training more closely simulates the movement patterns encountered in actual sports activities, thereby directly reinforcing the muscle groups and neuromuscular coordination associated with change-of-direction ability (Stern et al., 2020). Another reason may be the selection of Bulgarian split squats and reverse lunge plyometric jumps as movement patterns in the unilateral contrast training of this study. Both exercises share similar movement patterns with technical actions in basketball such as sliding defense and abrupt stops to evade defenders (Fisher and Wallin, 2014). Research indicates that incorporating Bulgarian split squats in resistance training is not only beneficial for enhancing core stability and lower limb muscle coordination but also aids athletes in making timely adjustments to changes in the center of gravity during directional changes or abrupt stops (Aguilera-Castells et al., 2019; De, Cantrell, and Schilling, 2014). Additionally, due to the stretch-shortening cycle mechanism of plyometric training, performing a rapid eccentric action followed by a rapid concentric muscle contraction allows athletes to store and utilize elastic energy and the stretch reflex in the lengthened muscle-tendon unit, thereby enhancing muscular activity (Turner and Jeffreys, 2010). Studies suggest that reverse lunge plyometric jumps can effectively develop the strength of the hamstring muscles under the same relative load, which is beneficial for improving technical actions that require significant hamstring involvement, such as landing and changing direction (Pappas et al., 2007). These factors may contribute to the superior effectiveness of unilateral contrast training in enhancing change-of-direction ability compared to bilateral contrast training. Furthermore, a wealth of literature has confirmed that unilateral training is superior to bilateral training in improving change-of-direction ability. Some scholars, through meta-analytic methods, summarized experimental changes in strength, jumping, linear, and directional speed measurements from unilateral and bilateral training in a total of 392 subjects (aged 16–26), finding that both training methods can improve change-of-direction ability, with unilateral training being more effective in enhancing the ability to change direction (Liao et al., 2022). Therefore, conducting 8 weeks of twice-weekly unilateral and bilateral contrast training is an effective method for improving change-of-direction ability, with unilateral contrast training being superior to bilateral contrast training.

### 4.3 Dynamic balance ability

An athlete’s dynamic balance significantly influences sports performance and the risk of injury. The YBT is considered the gold standard for assessing athletes' dynamic balance capabilities (Su, Yang, and He, 2021). Both contrast training modalities can significantly enhance the dynamic balance ability of basketball players. This improvement may be attributed to the fact that exercises such as the barbell back squat and the Bulgarian split squat both provide substantial stimulation to the core musculature, thereby improving core stability and enhancing joint stability and lower limb control (Cronin and Hansen, 2005). Upon analysis of the pre-and post-intervention differences in dynamic balance ability test indicators between the unilateral and bilateral contrast training groups, it was observed that prior to the experiment, the composite scores for both the unilateral and bilateral groups were below the 95th percentile. The composite score for the left leg was significantly higher than that for the right leg, and the discrepancy between the left and right legs was close to or exceeded the fifth percentile, indicating a notable difference in strength or balance between the support legs, which could increase the risk of sports injury (Su, Yang, and He, 2021). Post-intervention, both groups showed improvement, with the unilateral group demonstrating superior results to the bilateral group, albeit with varying degrees of enhancement. The unilateral group exhibited an 8.53% improvement in YBT (left) and a 13.8% improvement in YBT (right); in contrast, the bilateral group showed a 3.77% improvement in YBT (left) and a 4.72% improvement in YBT (right). The disparity in the enhancement of dynamic balance ability between the unilateral and bilateral groups may be due to task specificity. Basketball techniques, which involve a preponderance of unilateral movements such as jumping with one foot and shooting with one hand, could be a reason why the unilateral group showed better improvement in dynamic balance ability. Following the experiment, the unilateral group’s composite values for both the left and right legs exceeded the 95th percentile, and the balance discrepancy between the left and right legs was reduced from 6.98% to 3.06%. This indicates that the dynamic balance ability of both legs was improved, and the difference in dynamic balance was also reduced, thereby lowering the risk of sports injury. However, despite significant improvements, the bilateral contrast training group’s composite values for both legs still did not reach the 95th percentile, indicating a remaining higher risk of injury. This may be because unilateral strength training can produce adaptive responses through the nervous system, promoting cross-transfer. Cross-transfer refers to the phenomenon where unilateral muscle strength training results in increased strength in the contralateral homologous muscle (Green and Gabriel, 2018). Studies have shown that after unilateral training, the trained limb’s muscle strength can increase by 45.3%, and the untrained limb can also experience a 47.1% increase in strength, with the transfer effect depending on neural adaptability (Farthing et al., 2007). Therefore, when training the dominant side, the non-dominant side’s strength also improves, reducing the disparity in lower limb strength between sides and promoting the development of balance ability. Additionally, during unilateral contrast training, participants are required to maintain trunk stability. The unilateral force generation during the plyometric exercises, with single-foot takeoff and landing, is more unstable compared to double-footed actions, thereby enhancing neuromuscular control, which may also contribute to the unilateral group’s superior performance.

### 4.4 Limitation

This study only reflects the impact of unilateral and bilateral contrast training on athletes' lower limb athletic abilities through tests of explosive power, change-of-direction, and dynamic balance. It does not investigate other athletic abilities such as strength endurance, speed, or biomechanical performance including electromyography (EMG) analysis.

## 5 Conclusion

Compared to bilateral contrast training, unilateral contrast training has been found to be more effective in enhancing the performance of the non-dominant leg in jumping, sprinting abilities, change-of-direction capabilities, and dynamic balance. However, in terms of the CMJ, bilateral contrast training demonstrated superior results over unilateral contrast training. The improvements in maximal strength were similar between the two training modalities.

## 6 Application

Both unilateral and bilateral contrast training can serve as effective means to enhance the lower limb athletic abilities of college basketball players. The choice of training method should be based on specific training objectives.

## 7 Expectations

In the existing literature, there is a scarcity of research on unilateral and bilateral contrast training interventions lasting more than 8 weeks. Future studies could extend the duration of the intervention to explore the long-term effects of unilateral and bilateral contrast training on athletic abilities. This study did not categorize athletes by position for a detailed comparison. Subsequent research could delve into this aspect for a more refined analysis.

## Data Availability

The raw data supporting the conclusions of this article will be made available by the authors, without undue reservation.
